# The impact of weight cycling on gut microbiome richness and diversity in female rats

**DOI:** 10.14814/phy2.70828

**Published:** 2026-03-24

**Authors:** Madeline Wight, Chequita N. Brooks, Clare H. Scott Chialvo, Crystal A. West, Aline M. A. de Souza, Rachel M. Bleich

**Affiliations:** ^1^ Department of Biology Appalachian State University Boone North Carolina USA; ^2^ Louisiana Universities Marine Consortium Chauvin Louisiana USA; ^3^ Department of Medicine Georgetown University Washington DC USA

**Keywords:** diet restriction, food restriction, gut microbiome, microbial diversity, weight cycling

## Abstract

Female Fischer 344 rats were divided into control (access to food ad libitum) and weight cycling (60% diet restriction followed by ad libitum refeeding) groups. The weight cycles consisted of two weeks dietary restriction and three weeks refeeding for three cycles. Fecal microbiome samples were collected following the initial dietary restriction, the initial refeeding, and the final refeeding periods (and corresponding times in control rats). We observed significant differences in alpha diversity between fecal microbiomes following the initial dietary restriction and the final refeeding period. We additionally observed a significant recovery of alpha diversity following the first refeeding period that we did not observe following the third refeeding in the weight cycling group. Differences in relative abundances of taxa included a higher relative abundance of Bacillota (synonym Firmicutes) in the weight cycling group. Species richness of the weight cycling fecal microbiomes significantly decreased across the study period. Inguinal fat tissue was significantly lower in the weight cycling than ad libitum group, yet heart weight and postprandial HOMA‐IR were significantly higher. Together, these results suggest that repeated weight cycling from repeated periods of dietary restriction has adverse effects on host condition and microbial diversity, potentially leading to long‐term negative health outcomes.

## 1 INTRODUCTION

The gut microbiota plays a critical role in maintaining host health by regulating digestion, nutrient absorption, immune responses, and metabolic homeostasis. A diverse and balanced microbial community is a hallmark of a healthy gut ecosystem. However, drastic dietary changes—including restrictive eating or high‐calorie intake—can disrupt this balance, leading to dysbiosis and subsequent metabolic and inflammatory disturbances (Phuong‐Nguyen et al., 2024). Weight cycling, commonly referred to as yo‐yo dieting, is characterized by repeated episodes of weight loss followed by weight regain. This pattern, often driven by unsustainable dietary practices, has been associated with increased inflammation, altered gut barrier function, and enhanced energy efficiency—factors that may further exacerbate microbiome disruption (Phuong‐Nguyen et al., 2024).

Several animal studies have demonstrated that weight cycling can significantly impact gut microbial composition and diversity. For instance, weight cycling has been associated with decreased alpha diversity and shifts in relative microbial abundance, including a reduction in *Christensenella* spp. and *Lactobacillus reuteri* (Thaiss et al., 2016), and an increase in *Bacillota (synonym Firmicutes)* (notably *Ruminococcus*) and *Pseudomonadota (synonym Proteobacteria)* (e.g., *Desulfovibrio*) (Humblot et al., 2022; Kawashima et al., 2019). However, these studies primarily employed models cycling between high‐fat and low‐fat diets, focusing on obesogenic outcomes and diet composition effects.

In contrast, this study aimed to investigate the long‐term impact of weight cycling where lean animals were maintained on a standard grain‐based diet, with repeated cycles of severe caloric restriction (40% of ad libitum diet) followed by ad libitum refeeding. By isolating the effects of intermittent food restriction without the confounding influence of a high‐fat diet or changes in available nutrient profiles, this model allows for a clearer assessment of how the physiological effects of weight cycling influence gut microbiome dynamics and metabolic regulation over time.

## 2 RESULTS

### 2.1 Animal physiology changes with dietary restriction cycling

**FIGURE 1:**
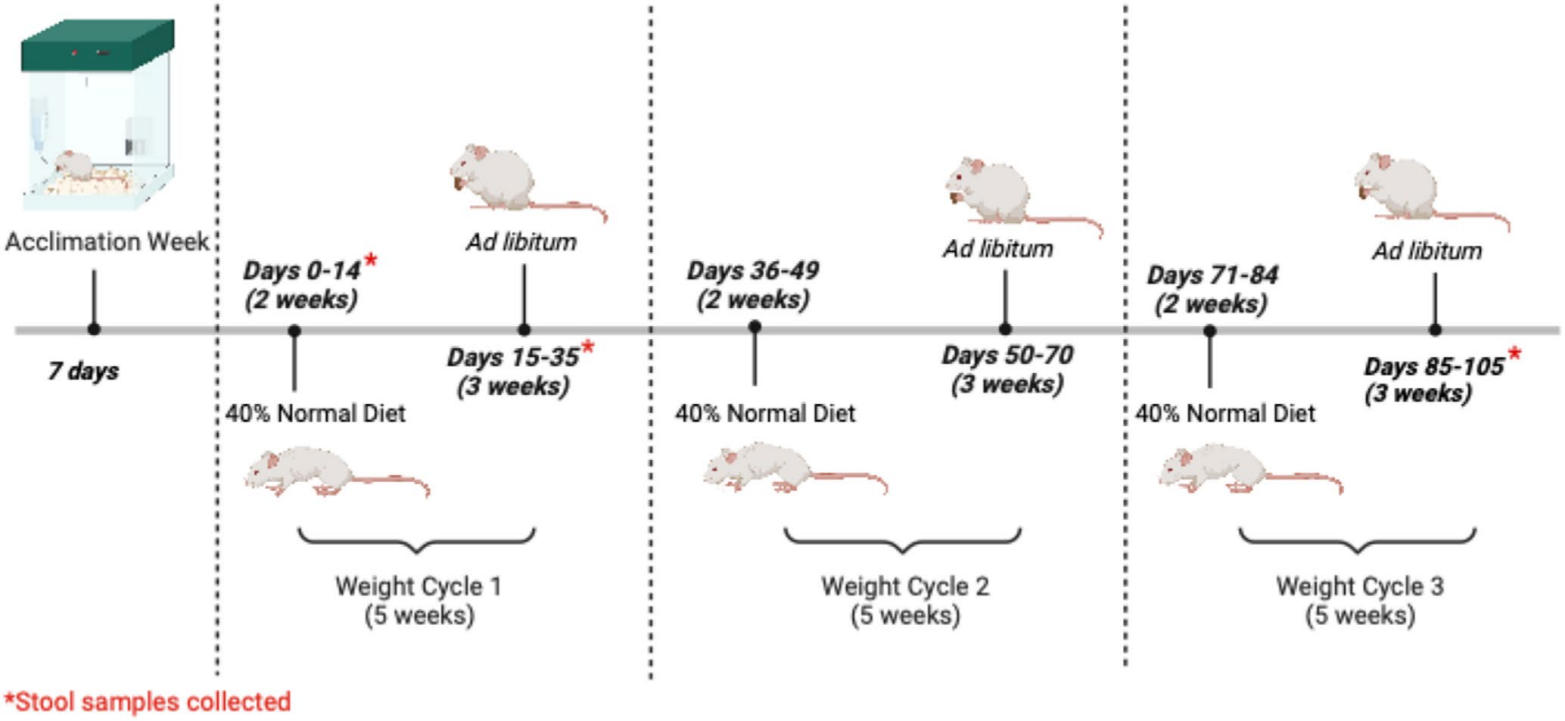
A graphical interpretation of the experimental timeline, with an emphasis on the food restriction treatment. Fischer F344 rats were allowed to acclimate to the experimental environment for 7 days before starting the experimental timeline. Days 0–14: rats were food restricted (60% reduction) in the weight cycling (WC) treatment or fed ad libitum. On day 14, fecal collections were taken from the ad libitum treatment; on day 15, fecal collections were taken from the WC treatment as they did not produce fecal matter on day 14. Days 15–35: all rats were fed ad libitum. Fecal collections were taken from all rats on day 35. The WC and ad libitum cycles were repeated three times. Fecal collections were taken from both groups on day 105.

**FIGURE 2:**
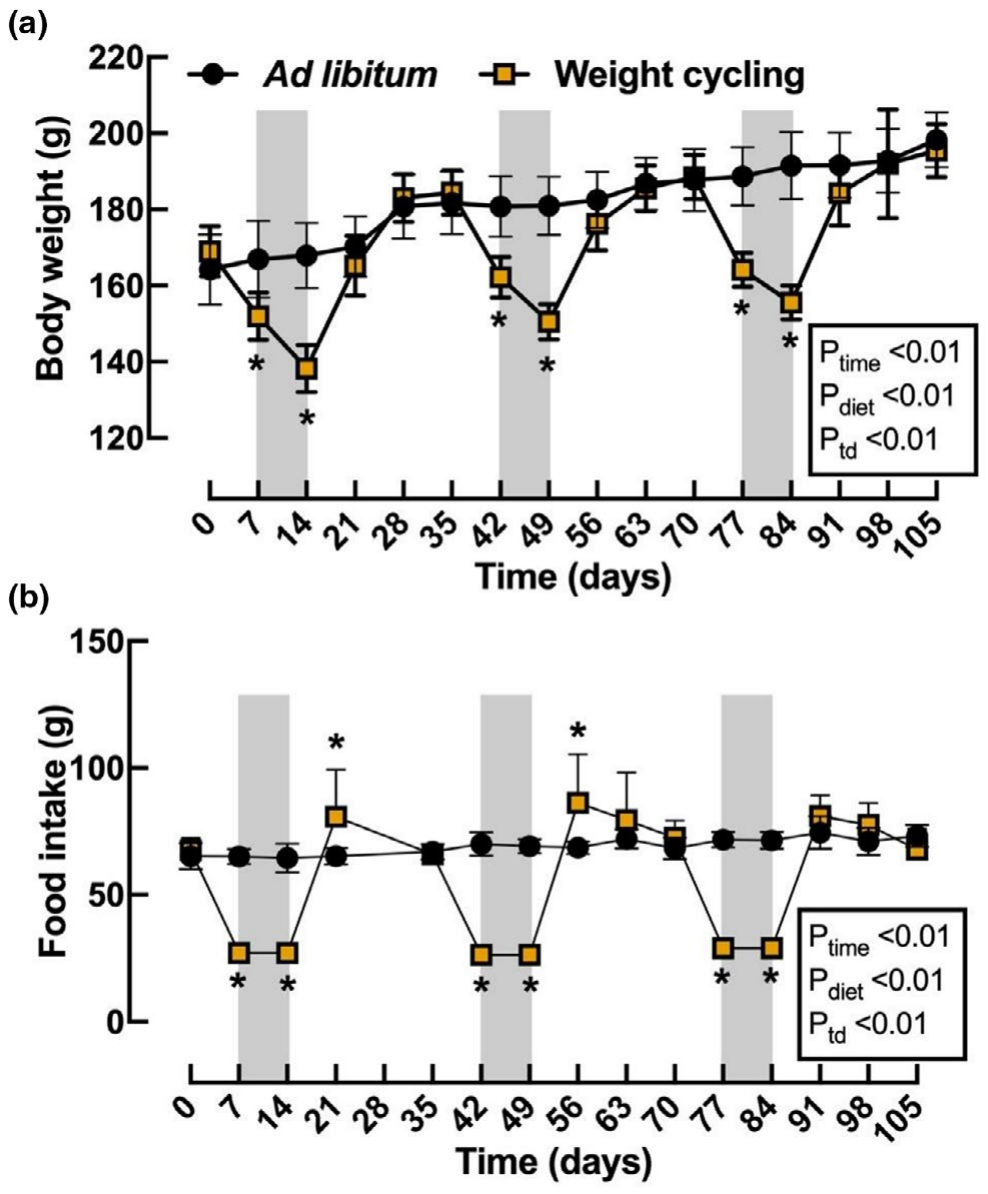
Effect of ad libitum and weight cycling (WC) treatment in body weight and food intake. The body weight evolution (a) and food intake (b) for ad libitum (black circle; *n* = 8) and weight cycling diet (yellow square; *n* = 8) are shown. Data were analyzed using 2‐way ANOVA for repeated measurement with the factors of time (P_time_), diet (P_diet_), and the probability of interaction between time and diet (P_td_); *p < 0.05 vs. Ad libitum, same time point by Bonferroni post hoc test. Values are expressed as the mean and standard deviation (SD).

Following the acclimation week (Figure 1), the rats placed in both the ad libitum and weight cycling (WC) groups showed similar body weight (BW) (Day 1: ad libitum 164 ± 9 g vs. WC 169 ± 7 g, *n* = 8, *p* > 0.99). However, after 2 weeks of food restriction, the WC rats lost around 18% of their initial BW, but it quickly returned to baseline levels following a return to an ad libitum diet. The second and the third rounds of food restriction caused similar BW loss in the WC group (Day 49: ad libitum 181 ± 8 g vs. WC 150 ± 4 g, *n* = 8, *p* < 0.01; Day 84: ad libitum 191 ± 9 g vs. WC 155 ± 4 g, *n* = 8, *p* < 0.01) (Figure 2a). The food intake was reduced by 60% from ad libitum across three cycles during the experimental period. We observed, following the initial food restriction period, that food intake (g) was significantly higher than the previously observed ad libitum intake (Day 21: ad libitum 65 ± 3 g vs. WC 81 ± 18 g, *n* = 8, *p* < 0.01) (Figure 2b). We again observed a statistically significant increase in food intake following the second food restriction period (Day 56: ad libitum 68 ± 3 g vs. WC 86 ± 19 g, *n* = 8, *p* < 0.01) (Figure 2b). Following the third food restriction period (Day 91), we did not observe a statistically significant food intake increase; however, there was a slight increase above the ad libitum baseline (Figure 2b).

On the euthanasia day (Day 105), the weight cycling treatment group was observed to have reduced inguinal fat and increased heart weight compared with the ad libitum treatment group (Table 1). However, we did not observe a significant difference between mean arterial blood pressure and heart rate between the two groups. The kidney, brown adipose tissue, brownness level of the brown adipose tissue, abdominal white adipose tissue, retroperitoneal, and parametrial white adipose tissue were also not significantly different between the two groups (Table 1).

**TABLE 1:** Effect of weight cycling on wet tissue weight, mean arterial pressure and heart rate.

Tissue	Ad libitum	Weight cycling	*p* value
	Mean ± SD (*n*)	Mean ± SD (*n*)	
Final BW	198 ± 7 (8)	195 ± 7 (8)	0.4513
Parametrial (g/100 g BW)	2.7 ± 0.5 (8)	2.6 ± 0.5 (8)	0.5213
Retroperitoneal (g/100 g BW)	0.96 ± 0.2 (8)	0.85 ± 0.2 (8)	0.3143
Inguinal (g/100 g BW)	1.23 ± 0.3 (8)	0.95 ± 0.2 (8)	0.0320*
Abdominal fat (%)	13.33 ± 2.02 (4)	16.24 ± 2.84 (4)	0.1460
BAT (g/100 g BW)	0.838 ± 0.01 (7)	0.080 ± 0.01 (8)	0.5725
BAT (% of Browness)	80.4 ± 8.9 (4)	75.7 ± 2.2 (4)	0.3461
Heart (g/100 g BW)	0.244 ± 0.008 (7)	0.256 ± 0.010 (8)	0.0261*
Kidney (g/100 g BW)	0.650 ± 0.02 (8)	0.639 ± 0.03 (8)	0.4356
MAP (mmHg)	84.5 ± 4.1 (8)	83.0 ± 3.2 (8)	0.4315
HR (BPM)	295.9 ± 38.6 (8)	314.0 ± 23.7 (8)	0.2774

*Note*: Tissue weight normalized to the rat's body weight on an ad libitum or weight cycling diet. Values are expressed as the mean ± standard deviation (SD).

* *p* < 0.05 vs. ad libitum, same tissue, by unpaired Student's *t*‐test.

**FIGURE 3:**
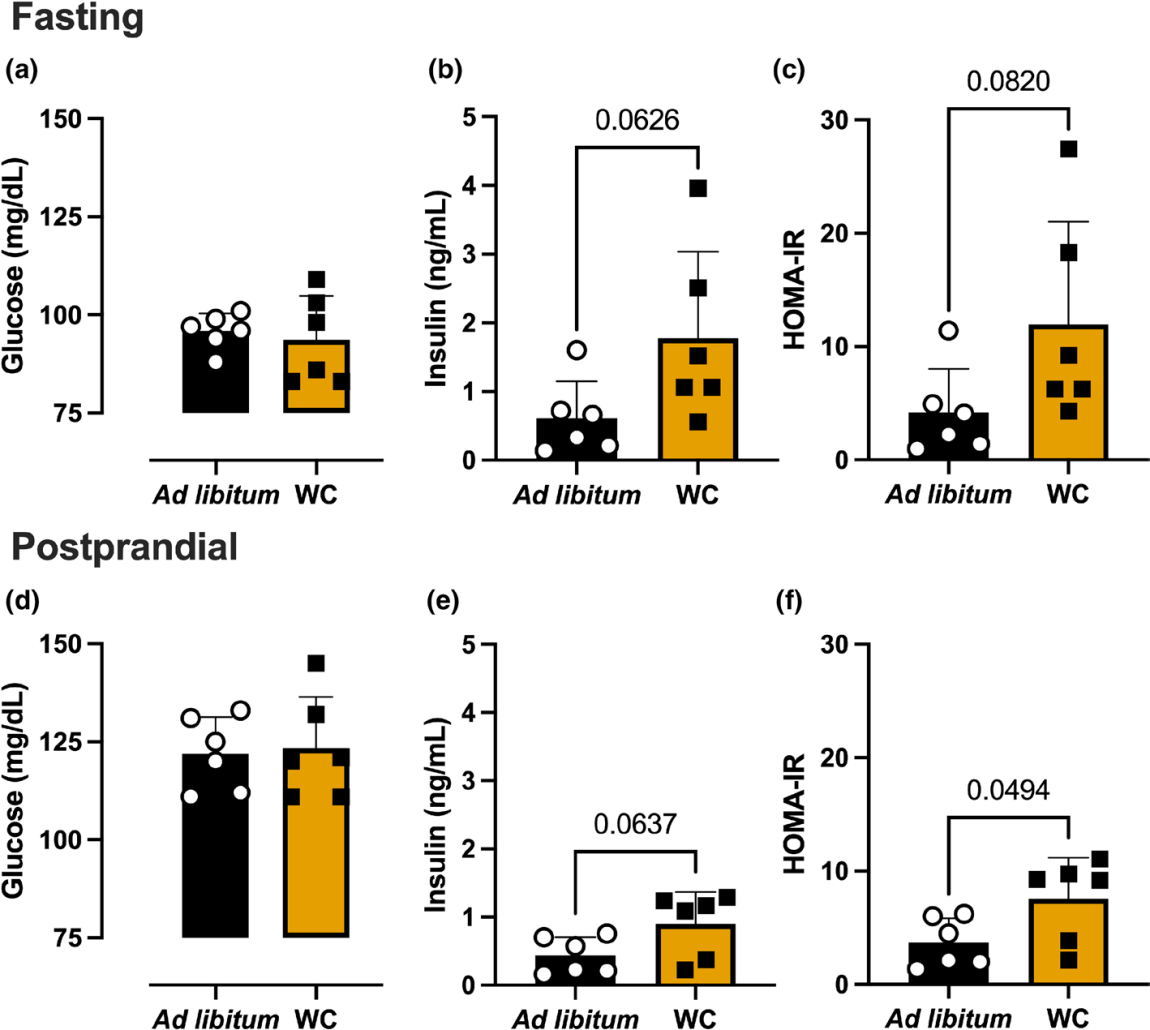
Effect of ad libitum (*n* = 6) and weight cycling (WC; *n* = 6) treatment in HOMA‐IR, insulin, and glucose. Shown are the homeostatic model assessments for insulin resistance (HOMA‐IR) (A & D), insulin (B & E), and glucose (C & F) for fasting and postprandial conditions. Data were analyzed using the Student's *t*‐test. Error bars are represented as standard deviation.

During data collection on Day 105, we found no significant difference between the two treatments' response in fasting glucose, insulin, or HOMA‐IR (Figure 3a–c). There is a slight increase in insulin and HOMA‐IR in the weight cycling treatment group compared to the ad libitum group. We did observe after a period of 2 h post‐food reintroduction, the weight cycling group showed a higher HOMA‐IR (ad libitum 3.7 ± 2.1 AU, *n* = 6 vs. WC 7.5 ± 3.6 AU, *n* = 6, *p* = 0.04) and a tendency for higher insulin levels (ad libitum 0.4 ± 0.3 ng/mL, n = 6 vs. WC 0.9 ± 0.5 ng/mL, *n* = 6, *p* = 0.06) (Figure 3e,f). There was no significant difference in glucose levels after food reintroduction between the two treatment groups (Figure 3d).

### 2.2 Fecal microbial diversity and richness

**FIGURE 4:**
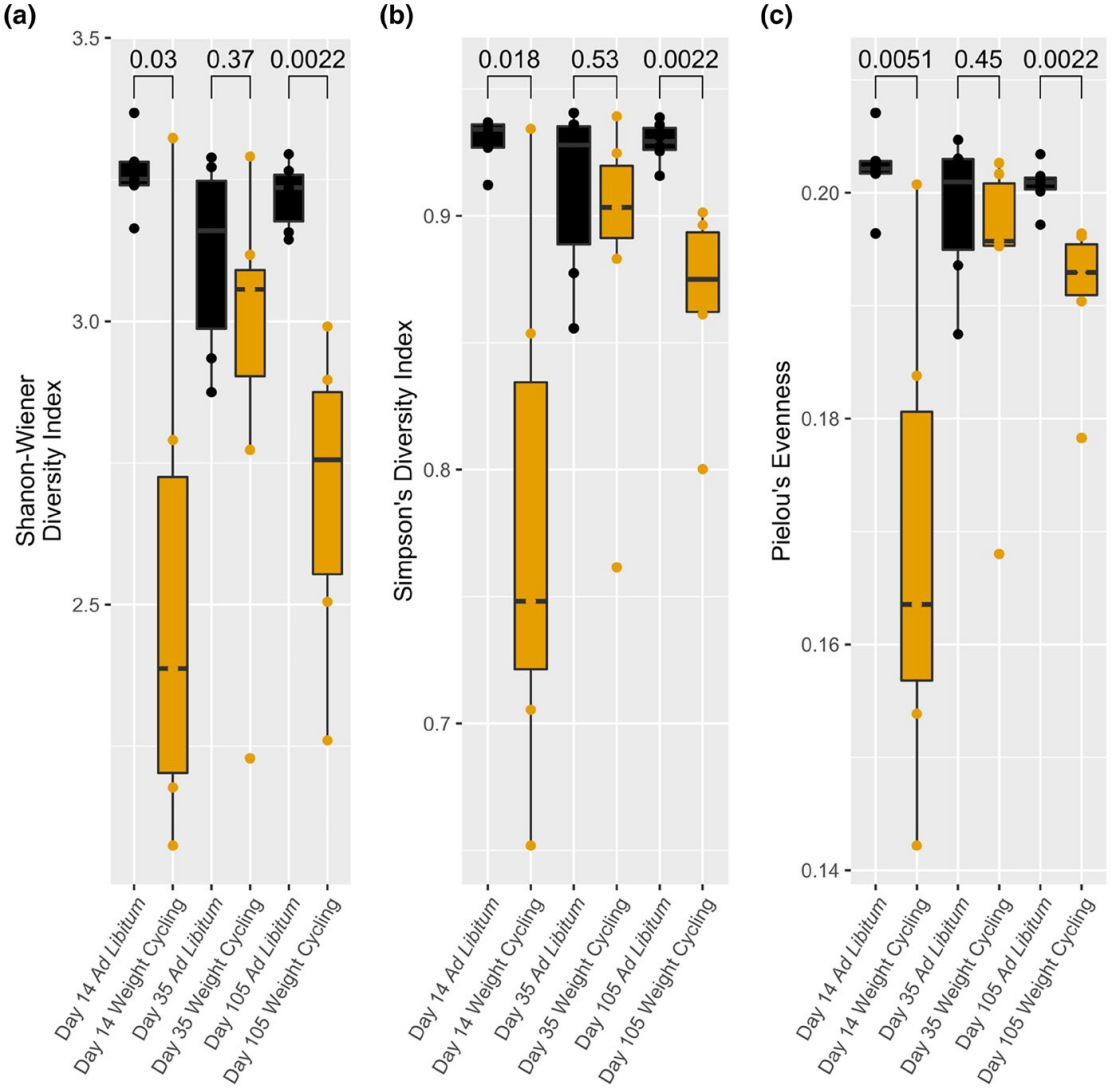
Ad libitum and weight cycling treatment groups of Fischer F344 rats' fecal microbiomes were quantified for the alpha diversity indices Shannon‐Wiener (a) and Simpson's (b). The evenness of the microbiomes was also quantified using Pielou's evenness index (c). At day 14, following the first two weeks of dietary restriction (40% of normal dietary intake), the alpha diversity of the weight cycling microbiomes was significantly lower than the ad libitum microbiomes (Shannon‐Wiener, *p* = 003; Simpson's, *p* = 0.018) as was the evenness (*p* = 0.0051). At day 35, following three weeks of ad libitum feeding for both treatments, the alpha diversity and evenness of the fecal microbiomes was not significantly different between the two treatment groups (Shannon‐Wiener, *p* = 0.37; Simpson's, *p* = 0.53; Pielou's, *p* = 0.45). Following the final ad libitum feeding following three treatment cycles, the two treatment groups did have significantly different alpha diversities and evenness (Shannon‐Wiener, *p* = 0.0022; Simpson's, *p* = 0.0022; Pielou's, *p* = 0.0022). Boxplots represent the median, first and third quartiles, and the whiskers extend to the furthest value no greater than 1.5*IQR from the first and third quartiles. IQR, interquartile range.

**FIGURE 5:**
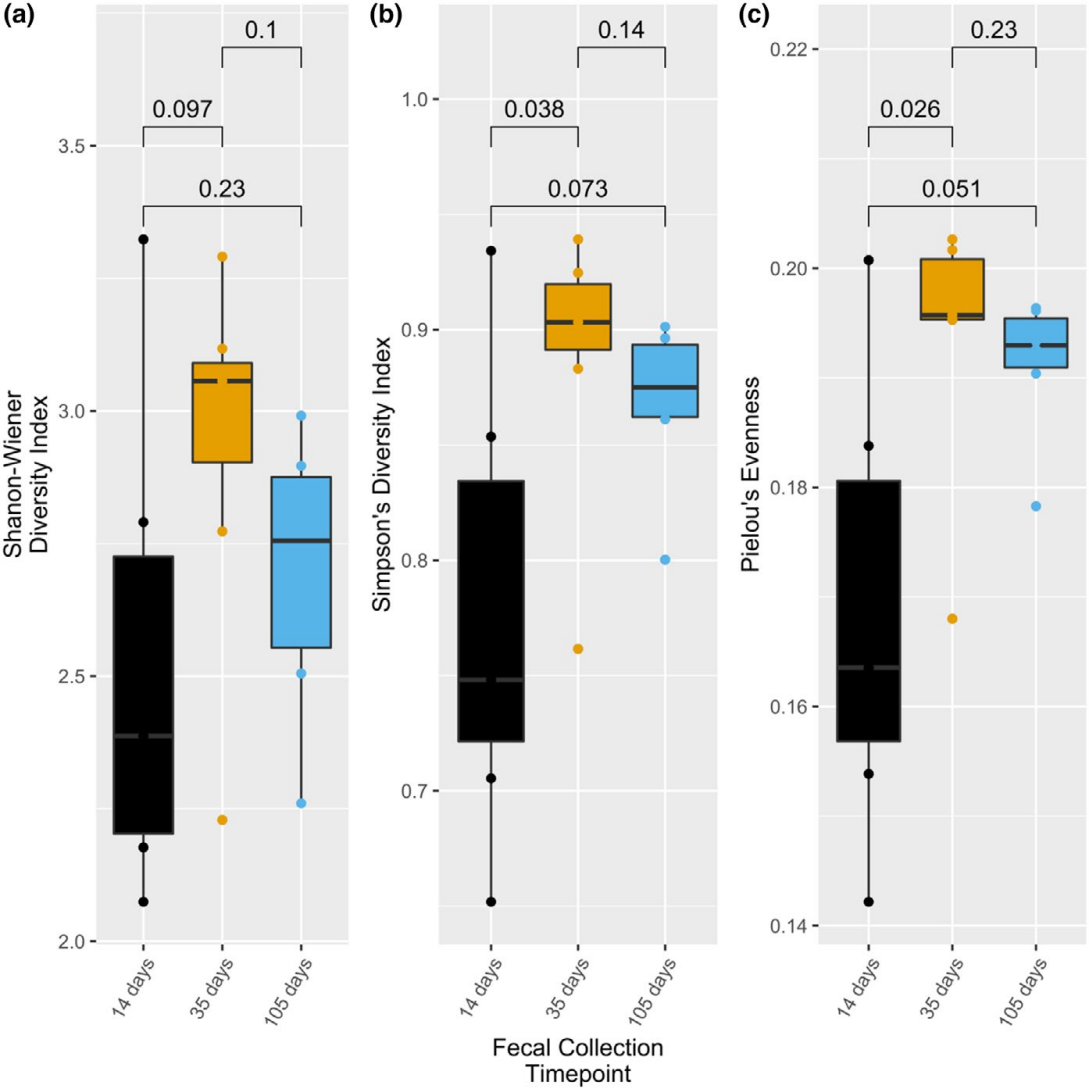
The weight cycling (40% of dietary intake cycled with ad libitum free feeding) treatment group of Fischer F344 rats' fecal microbiomes were quantified for alpha diversity at three timepoints, days 14, 35, and 105. Day 14 followed the initial dietary restriction period; day 35 followed two weeks of ad libitum diet; and day 105 followed a third period of ad libitum diet. Alpha diversity was measured using the Shannon‐Wiener diversity index (a) and Simpson's diversity index (b), and evenness was measured using the Pielou's evenness index (c). There was a significant difference in alpha diversity (Simpson's, *p* = 0.038) and evenness (Pielou's, *p* = 0.026) between days 14 and 35. No other significant differences were observed between fecal microbiome collection timepoints for the weight cycling treatment group. Boxplots represent the median, first and third quartiles, and the whiskers extend to the furthest value no greater than 1.5*IQR from the first and third quartiles. IQR, interquartile range.

We observed a significant difference in alpha diversity between ad libitum and weight cycling fecal microbiomes on Day 14, immediately following dietary restriction (40% of ad libitum baseline diet), and on Day 105, at the end of all three weight cycles. Alpha diversity was measured using the Shannon–Wiener diversity index (*p* = 0.03, *p* = 0.0022) and Simpson's diversity index (*p* = 0.018, *p* = 0.0022) (Figure 4a,b). We also observed a significant difference in community evenness using Pielou's evenness between ad libitum and weight cycling fecal microbiomes on Day 14 (*p* = 0.0051) and Day 105 (*p* = 0.0022) (Figure 4c). There was a significant difference in alpha diversity (Simpson's; *p* = 0.038) and evenness (Pielou's; *p* = 0.026) between Days 14 and 35 in the weight cycling fecal microbiomes (Figure 5). There was no significant difference for either alpha diversity or evenness between time points for ad libitum fecal microbiomes (Figure S1).

**FIGURE 6:**
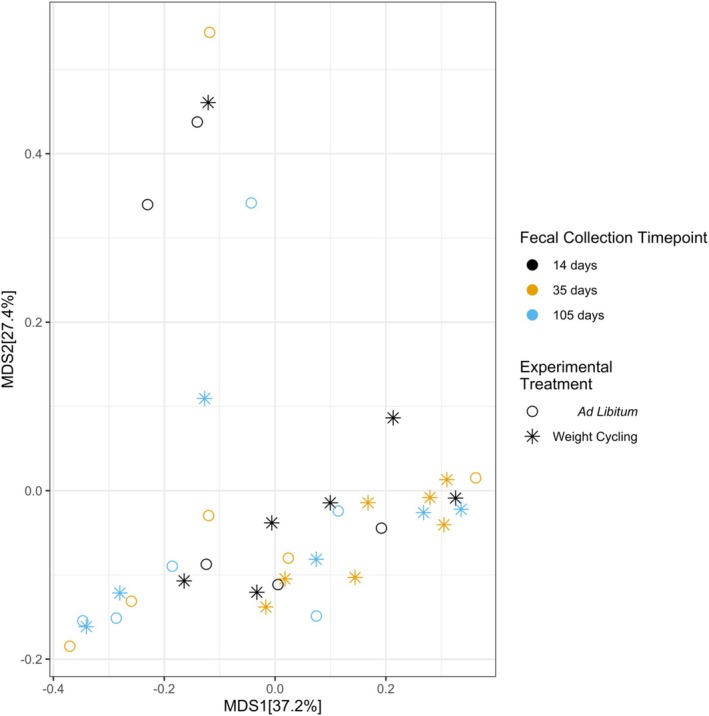
Using a Bray–Curtis dissimilarity analysis we calculated 37 eigenvalues to scale microbial communities based on their dissimilarity. We used the two highest eigenvalues (MDS1 and MDS2) to plot the microbial communities. Each community is represented by a data point, with shapes corresponding to the weight cycling (star) and ad libitum (circle) treatment groups. The color of each point corresponds to the fecal collection timepoints of 14 days (black), 35 days (yellow), and 105 days (blue). There were no observable trends between timepoints or treatment groups.

We observed no significant trend in beta diversity within or between the treatment group or timepoint microbiomes measured (Figure 6). The classification of fecal microbiomes over time within a single individual's samples varied considerably along the nonparametric eigenvalues MDS1, which explained 32.7% of the variation, and MDS2, which explained 27.4% of the variation between communities.

**FIGURE 7:**
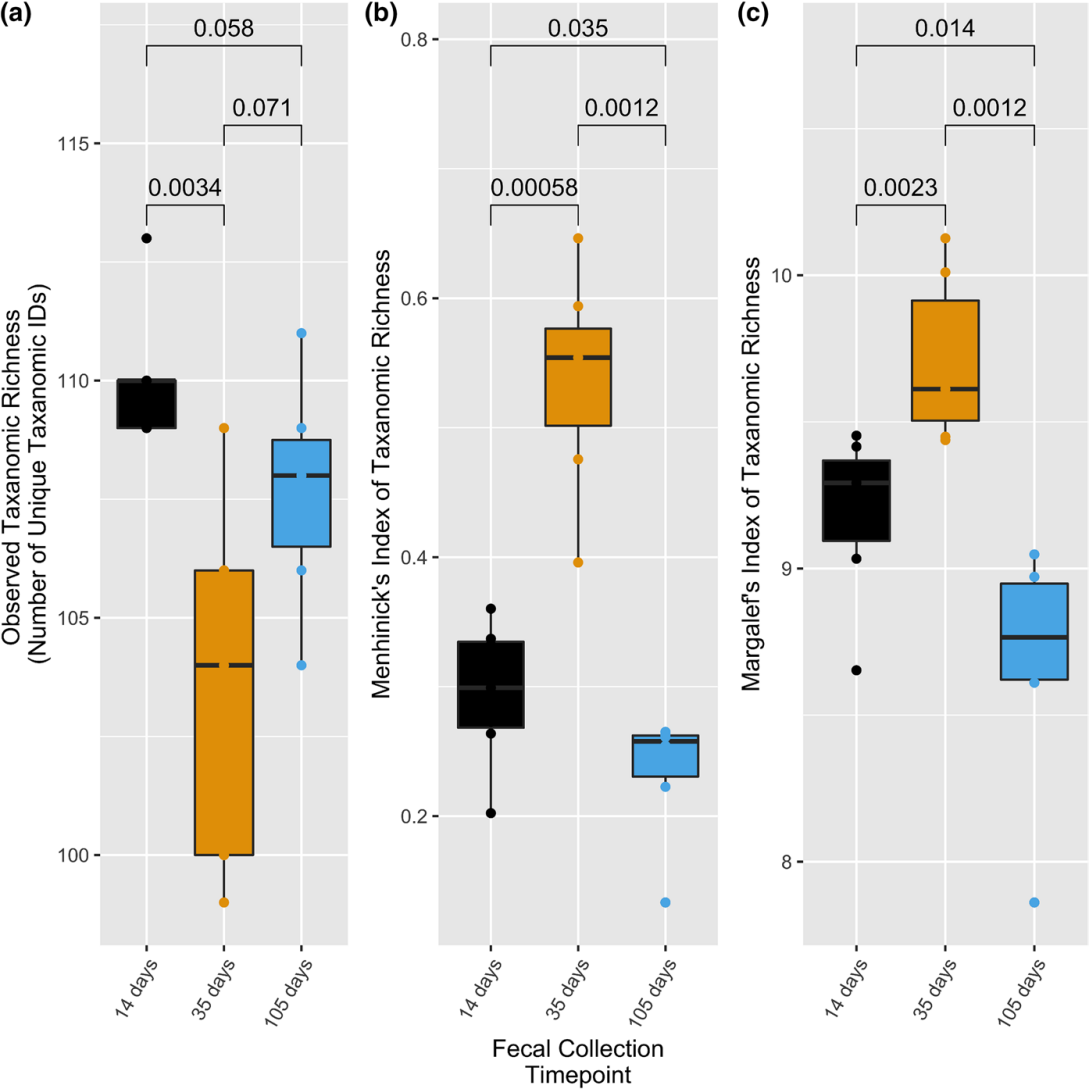
The weight cycling (40% of dietary intake cycled with ad libitum free feeding) treatment group of Fischer F344 rats' fecal microbiomes were quantified for species richness at three timepoints, days 14, 35, and 105. Day 14 followed the initial dietary restriction period, day 35 followed two weeks of ad libitum diet, and day 105 followed a third period of ad libitum diet. Species richness was quantified using observed taxonomic richness (a), Menhinick's index (b), and Margalef's index (c). There was a significant decrease in observed taxonomic richness between days 14 and 35 (*p* = 0.0034), however, both species richness indices indicated a significant increase in species richness during this time (Menhinick's, *p* = 0.0006; Margalef's, *p* = 0.0023). There was also a significant decrease in species richness between days 14 and 105 (Menhinick's; *p* = 0.035, Margalef's; *p* = 0.014) and between days 35 and 105 (Menhinick's; *p* = 0.0012, Margalef's; *p* = 0.0012). Boxplots represent the median, first and third quartiles, and the whiskers extend to the furthest value no greater than 1.5*IQR from the first and third quartiles (IQR = interquartile range).

We measured species richness using observed taxonomic richness, Menhinick's index of taxonomic richness, and Margalef's index of taxonomic richness. We observed a significant increase in species richness between Days 14 and 35 in the WC fecal microbiomes (Menhinick's; *p* = 0.0006, Margalef's; *p* = 0.0023) (Figure 7). There was also a significant decrease in species richness between Days 14 and 105 (Menhinick's; *p* = 0.035, Margalef's; *p* = 0.014) and between Days 35 and 105 (Menhinick's; *p* = 0.0012, Margalef's; *p* = 0.0012). We did not observe a difference in species richness between Days 14 and 105 in the ad libitum fecal microbiomes (Menhinick's; *p* = 0.13, Margalef's; *p* = 0.082) (Figure S2). However, there was a significant increase in species richness in the ad libitum fecal microbiomes between Days 14 and 35 (Menhinick's; *p* = 0.0043, Margalef's; *p* = 0.0043) and a significant decrease between Days 35 and 105 (Menhinick's; *p* = 0.0022, Margalef's; *p* = 0.0022) (Figure S2).

### 2.3 Fecal microbiome relative abundance

**FIGURE 8:**
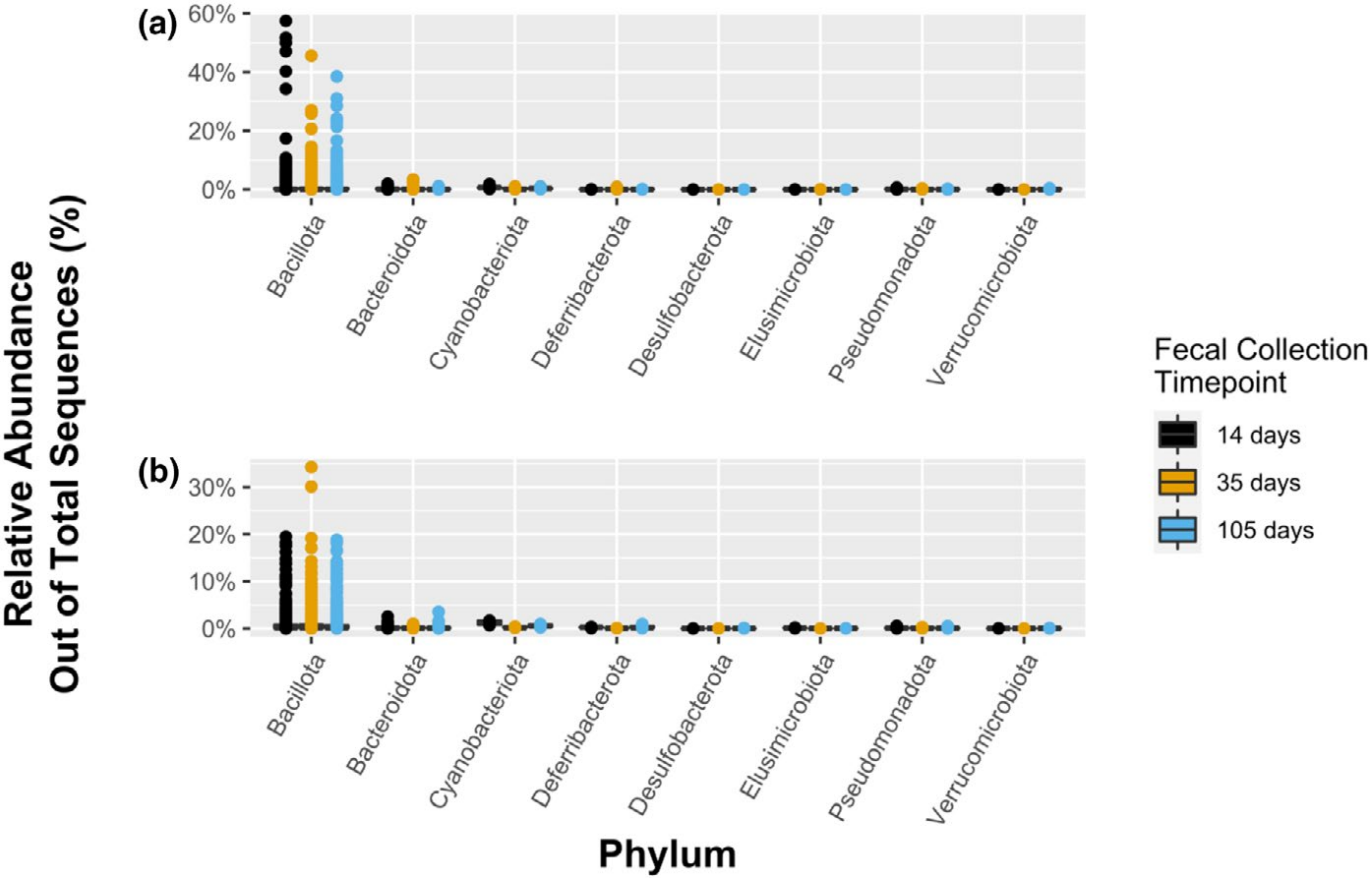
The relative abundance of 16S amplicons from the (a) weight cycling (40% of dietary intake cycled with ad libitum free feeding) and (b) ad libitum treatment groups of Fischer F344 rats' fecal microbiomes were calculated at the phylum level for timepoints 14 days (black), 35 days (yellow), and 105 days (blue). There was no significant difference in relative abundances in either treatment group for any of the above phyla over time. However, there was an observable decrease in highest relative abundance Bacillota (synonym: Firmicutes) OTUs over time in the dietary restriction microbiomes.

The highest relative abundance phylum observed in both the WC and ad libitum fecal microbiomes was the Bacillota (synonym: Firmicutes; 57% and 34%, respectively), followed by the Bacteroidota (synonym: Bacteroidetes; 3.4% and 3.5%, respectively). There was no significant difference in Bacillota relative abundance between timepoints for either treatment group; however, there was a trend toward a reduction in Bacillota relative abundance over time in the highest relative abundance OTUs in the WC fecal microbiomes that was not mirrored by an increase in Bacteroidota (Figure 8).

Highest relative abundance families in the phylum Bacillota from the WC microbiomes were Lactobacillaceae, Clostridiaceae, and Lachnospiraceae. Highest relative abundance families in the phylum Bacteroidota from the WC microbiomes were Muribaculaceae, Bacteroides, and Prevotellaceae.

## 3 DISCUSSION

The present study evaluated the impact of 3 cycles of 60% food restriction from ad libitum followed by weight recovery on the microbiome and basic physiological parameters of Fischer F344 rats. Physiological parameters included blood pressure, heart rate, and insulin resistance. Our results show that every cycle of food restriction affected body weight with the same intensity as observed in previous studies with rats under the same diet and duration (de Souza et al., 2015; de Souza et al., 2018). The number of cumulative food restriction cycles did not affect the rate of body weight loss observed. In our study, the rats recovered the lost body weight in 1 week of ad libitum diet, and we did not observe a higher body weight gain at the end of the third cycle of food restriction to ad libitum. In a similar study using female Fischer rats, but with only one round of low food intake, at the end of the 3‐month refeeding period the rats had the same body weight as the controls (de Souza et al., 2020; Thaiss et al., 2016).

We observed that rats in the WC treatment group showed a statistically significant compensatory overeating behavior during the first week of the ad libitum diet following the first cycle of food restriction and return to ad libitum; however, this did not happen in the third cycle. This finding was opposite to what Rosenbaum et al. (2019) reported. They reduced rats' food intake by 50% until they lost 20% of their initial body weight, followed by an ad libitum period across four cycles. The “cycler” rats had lower body weight at the end of the study and lower food intake when compared to ad libitum controls but had no statistical difference from starting condition (Rosenbaum et al., 2019). The difference in food intake observations between these studies could be attributable to the method of data collection. Rosenbaum et al. averaged food intake data for periods of approximately 50 days (2019), whereas we calculated food intake weekly. It has also been observed in human study participants following a single period of semi‐starvation that there is a transient higher energy intake even after 100% of the lost body weight is recovered, as well as an observed higher fat deposition (Dulloo, 2021). We did not see an effect on the total fat or brown adipose tissue in our model, however, the inguinal fat was lower in the WC rats versus ad libitum controls. We may not have observed a statistically significant increase in total fat or brown adipose tissue due to the limited time for the rats to recover all fat lost and make extra adipose tissue during weight cycles. Previously our group has observed that it takes at least 40 days for the rats to have a significant deposition of adipose tissue after a single period of food restriction (*data not published*). Still, the change in white adipose tissue (inguinal fat) is an interesting phenomenon, as the fat pad is associated with the most developed nipples (inguinal) of female rats (Masso‐Welch et al., 2000) and is therefore strongly associated with sex hormones (e.g., estrogen) (Chusyd et al., 2016).

The blood pressure and heart rate were observed to be similar between the WC and ad libitum treatment groups following the 105‐day study period. This finding is similar to self‐reporting studies in human weight cyclers, showing no difference in systolic or diastolic blood pressure (Kakinami et al., 2020). In animal models, we know that one cycle of food restriction reduces the blood pressure during the food restriction period, but it goes back to normal after a week of ad libitum feeding, staying at that level for 3 months (de Souza et al., 2020; Thaiss et al., 2016). However, in a weight cycling protocol using obese spontaneously hypertensive rats, after three cycles of 12 days under a very low‐calorie diet followed by four weeks ad libitum refeeding, the blood pressure was higher than the initial baseline (Ernsberger et al., 1998). Not only is the blood pressure affected by diet cycling but also the heart rate and heart size. We observed WC rats had a significantly higher heart weight when compared to ad libitum control rats. This is an initial observation that needs to be further investigated, but it could be an indication of present or future heart disease. It has been previously observed that weight cycling in obese human populations is a risk factor for cardiometabolic disease (Montani et al., 2015) and has also been associated with poor prognosis in the diabetic population (Kaze et al., 2022).

The low‐calorie diets employed in studies are variable throughout the literature, and it is hard to compare our findings with models that do not use the same metric of dietary restriction. This is further complicated by the variable periods of time and differing initial body weights and ages of rodent models. We hypothesize that this may lead to seemingly contradictory findings in the literature, which may result from variable responses to these multiple confounding variables. In a review paper about weight cycling and animal models, Reed and Hill found 7 publications saying that weight cycling does not affect insulin resistance, but one study found that cycling increases fasting insulin levels (Reed & Hill, 1993). The models were diverse: obesity‐prone rat versus Sprague Dawley, male vs. female, fasting or restrictive diet, 2 or 3 weeks of diet followed by 2–5 weeks of refeeding. The variability in study design has only increased since 1993. The study that found a significant impact on fasting insulin used female Sprague Dawley rats under a restrictive diet to make them lose 25% of their body weight (Reed et al., 1988). This finding was similar to our observations where we saw higher levels of postprandial insulin with a trend for fasting insulin. Contributing to this finding, in a study of self‐reporting weight history, women and men with a history of weight cycling showed worse HOMA‐IR when compared with persons who reported a stable weight (Kakinami et al., 2020).

Insulin resistance has been increasingly associated with alterations in the gut microbiome.

A diverse and balanced microbial community within the gastrointestinal tract is believed to enhance insulin sensitivity, and drastic dietary changes, including restrictive eating or high‐calorie intake, can disrupt this balance, leading to dysbiosis and subsequent metabolic and inflammatory disturbances (Phuong‐Nguyen et al., 2024). Our study yielded results suggesting that fecal microbial communities are increasingly sensitive to periods of dietary restriction as they are repeated. We found that there was a significant increase in alpha diversity between the end of the first period of dietary restriction (0–14 days) and the end of the first period of re‐feeding at ad libitum levels (15–35 days) in the WC treatment group. However, there was no longer a significantly higher alpha diversity at the end of the third re‐feeding at ad libitum levels (85–105 days) from the Day 14 measurements. These results suggest that the periodic dietary restriction had a cumulative impact on the microbial community diversity over the course of three periods (Days 0–14, 36–49, 71–84). The cumulative effect of periodic dietary restriction was further exemplified in the decrease in species richness, with a significant decrease in species richness between collections on Days 14 and 105. The impact of cyclical dietary restriction may therefore have important implications for overall host health. Modeling of gut microbiomes for optimized colonization resistance suggests that low richness, high diversity populations can better resist colonization than high richness, high diversity populations (Zhu & Momeni, 2024). While we do not have a Day 0 fecal collection, we can ascertain that the Day 35 ad libitum sample from the WC rats had both high diversity and high richness. This may have made the rat gut microbiomes more prone to colonization by microbial taxa with the potential for negative host health impacts according to the previously modeled predictions. The failure to recover at Day 105 may additionally be due to a variety of host factors including alterations in the quantity of bile salts present in the gut (Schubert et al., 2017), increases in inflammatory cytokines (Baskurt & Yardimci, 2024), or the depletion of obligate anaerobic species (Ecklu‐Mensah et al., 2022).

The literature on the influence of weight cycling diets on gut microbial diversity is similarly murky. In a study of physique competitors between 19 and 40 years of age, microbial diversity (Shannon) was not significantly different between pre‐ and post‐diet (following a 23‐week period of weight loss) (Driuchina et al., 2025). However, the gut microbiome was observed to be significantly *more* diverse and increased in richness following the 23‐week recovery (weight regain) period compared to both the pre‐ and post‐diet diversity (Driuchina et al., 2025). In a meta‐analysis of 47 weight loss intervention trials in adults designated as overweight or obese (81% female), dietary restriction resulted in an increased alpha diversity (Koutoukidis et al., 2022). Alpha diversity standardized mean difference had a positive relationship with purported weight loss, with greatest weight loss (loss of 20–50 kg) having the greatest increase in alpha diversity (Koutoukidis et al., 2022). This meta‐analysis did not, however, interrogate the influence of weight cycling on the microbiome, likely due to a lack of data collection when weight regain occurred. In an example of the benefits of using controlled rodent dieter populations, the study by Fouesnard et al. interrogated the effects of a two‐cycle weight *gain* (or overweight) period followed by diet maintenance and weight loss in C57BL6/N male mice (2025). Fecal samples were collected from the caecal content following the end of the second weight loss period and 16S rDNA amplification found a significantly *lower* number of observed species in the weight cycling mice and no significant difference in Shannon diversity compared to controls (Fouesnard et al. 2025). In a similar study of weight gain followed by weight loss where multiple timepoints were observed from C57BL/6 male mice, alpha diversity (Chao1) is significantly *lower* during the induced obesity stage and after weight loss compared to controls (Thaiss et al., 2016).

We did not observe any trends in the beta diversity measurements of the fecal microbial communities. Our Bray–Curtis dissimilarity analysis indicated that individuals' microbiomes shifted across both MDS1 and MDS2 over the 105‐day observation period. Activity level, in the form of exercise quantification, has been found to have varied impacts on the composition and diversity of gut microbiomes (Brooks et al., 2023). We posit that this or other behavioral differences might lead to differences in microbial community response to dietary restriction. However, there is lacking literature tying activity level and dietary restriction together, especially in regard to microbiome responses. It is furthermore unclear how cyclical dietary restriction might influence the functioning of the microbiome and host immune system in the event of infection or other perceived stressors. Preliminary work has suggested that cyclical dietary restriction that leads to adipose tissue gain and loss can trigger immune responses to adipose tissue that are increasingly inflammatory (Anderson‐Baucum et al., 2014), however, to our knowledge, these hypotheses remain untested.

The fecal microbiomes of the dietary restriction treatment had a much higher relative abundance of Bacillota than the ad libitum treatment (57% and 34%, respectively). This result mirrors those of another study, where five‐week‐old female C57BL/6 mice were fed high fat diets, followed by a standard diet, and then fed a high fat diet again and had an increase in Bacillota relative abundance (Kawashima et al., 2019). In a study of human gut microbiomes, both under‐ and over‐eating relative to weight maintaining energy needs led to a shift in the dominant phyla, with Bacillota increasing relative to decreases in Bacteroidota, which had a significant correlation with nutrient absorption (Jumpertz et al., 2011). A study of teenage human girls diagnosed with anorexia nervosa additionally found that the production of the protein zonulin, which increases the permeability of tight junctions between intestinal cells (Fasano, 2012), had a significant positive correlation with the phylum Bacillota and organisms in the genera *Escherichia* and *Shigella* (Soltysova et al., 2025). Bacillota were also found to have a significant negative correlation with brain‐derived neurotrophic factor (BDNF) (Soltysova et al., 2025). While the results of various studies such as ours have found these microbiome shifts relative to host physiology, the field needs further study into the metabolism (microbial activity) in the host gut and how it may influence gut permeability and immunity (Phuong‐Nguyen et al., 2024). Work in rodent models *has* shown that the secretion of neurotrophic factors, such as BDNF, can be modulated by members of the gut microbiome (e.g., *Bifidobacterium breve* DPC 6330, phylum: Actinomycetota (synonym: Actinobacteria)) and lead to host health outcomes that mimic models of early‐life stress and irritable bowel syndrome (O'Sullivan et al., 2011). Other microbial metabolites, such as short‐chain fatty acids (many producers are Bacillota members), have more complex and varied impacts on host physiology that remain unresolved (Joyce & Clarke, 2024). Recent advances in parsing metagenomic data for gut microbial metabolisms (e.g., Andreu et al., 2023) demonstrate that there is still much to be learned from mechanistic studies of targeted taxa relative to host physiological response.

## 4 CONCLUSIONS

We analyzed the impact of weight cycling caused by three rounds of severe caloric restriction on the fecal microbiome. Body weight in Fischer F344 rats dropped by approximately 18% during the three periods of dietary restriction that rebounded during ad libitum refeeding. This led to reduced inguinal fat and increased heart weight, but no change in mean arterial blood pressure, heart rate, or other adipose tissues. No significant difference in fasting glucose, insulin, or HOMA‐IR was observed, except for higher HOMA‐IR 2 h after rats were allowed access to food following a fasting period. With these changes in physiology, we observed alterations to the fecal microbiome with weight cycling over time. Alpha diversity was significantly reduced in the fecal microbiomes of the weight cycling group at the end of the three weight cycles, which correlated with reduced microbial species richness and evenness. This suggests that gut microbial communities suffer from decreased efficacy in recovery during repeated weight cycling events. We observed a decrease in alpha diversity in the WC compared to ad libitum treatments, and a subsequent rebound following the first cycle of refeeding at ad libitum levels. However, the recovery was diminished following the third cycle of dietary restriction and subsequent refeeding. The WC treatment fecal microbiomes also had higher relative abundance of Bacillota compared to the ad libitum control. These results demonstrate that repeated weight cycles, where weight is dramatically reduced in otherwise healthy animals, can lead to changes in host physiology and the fecal microbiome that could impact host health longer term.

## 5 METHODS

### 5.1 Animals

Animals were maintained in the Georgetown University animal facility in compliance with institutional guidelines and the Guide for the Care and Use of Laboratory Animals, National Research Council (NRC) Publication, 2011 edition. All animal protocols were approved by Georgetown University's Institutional Animal Care and Use Committee, and experiments were carried out according to institutional guidelines (protocol no. 2016‐1182). Experiments were conducted using 12‐week‐old female Fischer F344 rats (Envigo) single‐housed the whole experimental period. Once acclimated to the environment, food intake and body weight were determined for one week to establish a baseline. Rats were divided and randomly assigned to a control group (ad libitum) or assigned to the weight cycling (WC) experimental group (dietary restriction cycling over three intervals). Dietary restriction cycling consisted of 14 days of food provision of 40% of the typical diet followed by an ad libitum re‐feeding phase for 21 days, repeated three times (Figure 1). All rats were fed a nutritionally complete food (Teklad Global 19% Protein Extruded Rodent Diet, Madison, WI). Fecal samples were collected at four time points: Day 14 of dietary restriction or on the first day of the first refeeding (WC rats did not produce fecal samples on day 14), after 3 weeks of ad libitum refeeding (Day 35), and at the end of the third cycle of dietary restriction and refeeding (day 104). Fecal frozen samples were shipped on dry ice to Appalachian State University.

### 5.2 Animal physiology measurements

On Day 104, the rats from both the ad libitum and WC treatments were fasted overnight. On day 105, blood fasting glucose was measured by pinching the tail with a lancet and collecting a blood drop for measurement using a OneTouch Ultra Mini glucometer (LifeScan, Malvern, PA). Blood fasting insulin was measured using ELISA following manufacturer's instructions (Rat Insulin Kit, Crystal Chem, Elk Grove Village, IL Catalogue #90060). After baseline blood glucose and insulin collection, all rats were fed ad libitum for 30 min. After a waiting period of 2 h, blood glucose and insulin were again collected for the post‐prandial time point. The HOMA index was calculated for both fasting and post‐prandial time points as previously described (Knopp et al., 2019).

At the end of the third cycle of dietary restriction and refeeding (Day 106), we determined rat blood pressure using femoral catheterization as described previously (de Souza et al., 2018). Briefly, the rats were anesthetized with 2.5% isoflurane at 1 L/min oxygen and a polyethylene catheter (PE50) was inserted into the femoral artery. The PE50 was connected to a pressure transducer (MLT0699; ADInstruments, Colorado Springs, CO). The data were converted in PowerLab (ADInstruments, Colorado Springs, CO), and blood pressure was calculated from a 20‐min interval using the Chart software v. 8.0 for Mac (ADInstruments, Colorado Springs, CO).

### 5.3 Magnetic resonance imaging (MRI)

On Day 103, magnetic resonance imaging (MRI) was performed in the Georgetown‐Lombardi Preclinical Imaging Research Laboratory (PIRL) on a Bruker 7T/30 MR Scanner run by an Avance NEO system and ParaVision 360 software. Prior to MRI, the rats were anesthetized with 1.5% isoflurane and placed on a custom manufactured stereotaxic device with built‐in temperature and cardio‐respiratory monitoring (ASI Instruments, Warren, MI) previously described (Coia et al., 2021). For fat imaging, a 40 mm Bruker rat body volume coil was used.

Nuchal fat imaging was performed with a T2‐weighted RARE protocol in the sagittal orientation with TR: 2800 ms, TE: 15 ms, matrix: 256 × 256, and FA: 180. Analysis of neck fat entailed quantifying the relative mean intensity of brown fat (BF) and white fat (WF). For this, BF and WF regions of interest (ROI) were selected on three separate slices and corresponding mean intensities were averaged for each rat. Quantification of fat depots in the imaging datasets was performed by thresholding and voxel‐counting with Fiji software version 2.14.0/1.55f (NIH) as described previously (Liggett et al., 2022). Briefly, a maximum intensity projection algorithm of the image datasets was used with an intensity threshold intended to segment only the visceral fat of the abdominal area.

### 5.4 Statistical interpretation of physiology data

The physiological data were analyzed by Prism software (v. 10.0, GraphPad, La Jolla, CA). Tissue weight, insulin, glucose, blood pressure, and heart rate were analyzed initially by Shapiro–Wilk normality test and, following normality, the data were analyzed by Student's *t*‐test nonpaired. Time course was analyzed by 2‐way ANOVA for repeated measurement followed by Bonferroni post hoc. Significance was defined by *p* < 0.05.

### 5.5 Fecal DNA isolation & minION 16S sequencing

Fecal DNA extraction was facilitated using the QIAamp PowerFecal Pro DNA isolation kit (Catalogue #51804, Qiagen, Germantown, MD) following the manufacturer's protocol. A NanoDrop Microvolume Spectrophotometer was used to quantify DNA concentration before library preparation. Library preparation was performed using a 16S Barcoding Kit 1–24 (Oxford Nanopore Technologies, Oxford, UK). Samples are divided into four amplicon sequencing libraries for 16S rDNA metabarcode analysis following the manufacturer's protocol. Sequencing was performed using the MinION Mk1B sequencing device (Oxford Nanopore Technologies, Oxford, UK). Each library was sequenced for ≥20 h prior to being manually concluded. FASTQ files were generated after sequencing using the Guppy basecaller (Wick et al., 2019).

### 5.6 Taxonomic assignment and data curation of 16S rDNA amplicon sequences

The FASTQ files were appended with the library number on each sequence header, and the FASTQ sequences were concatenated into a single FASTQ file. Using the “emu abundance” command, the sequences were classified in EMU (Curry et al., 2022) using the SILVA reference database v. 138.1 (Quast et al., 2013). The taxonomic assignment took a total CPU time of 47,7802.331 s and assigned 89,411,528 sequences across 31 samples. Two output files from EMU were used for downstream applications (rel‐abundance‐threshold‐0.0001.tsv, read‐assignment‐distributions.tsv). The “read‐assignment‐distributions.tsv” output file was reformatted and the data were pared down using awk (Aho et al., 1979). The annotated code is available in Supplemental File—Appendix S1.

### 5.7 Statistical analysis of microbial community diversity

Statistical analyses were completed in R v. 3.5.2 (R Core Team, 2018). The “rel‐abundance‐threshold‐0.0001.tsv” output file from EMU, a user‐generated map file (metadata), and the awk generated files were imported into the R environment. The EMU output file was reformatted to separate lineages into separate levels of classification using the packages dplyr v. 1.0.2 (Wickham et al., 2020) and tidyr v. 1.1.2 (Wickham, 2020b). The taxa count file was reformatted into a matrix using tidyr, and datasets were merged using dplyr. The matrix count file was rarefied to the lowest number of reads (*n* = 15,931) using the packages vegan v. 2.5.6 (Oksanen et al., 2019) and tibble v. 3.0.3 (Müller & Wickham, 2020). The matrix count file was rarefied for this data set as there was a large disparity between the number of reads in each sample library, which could influence alpha diversity indices, which are sensitive to trailing tails (many low abundance taxa). The alpha diversity indices Shannon–Wiener, Simpson's, and Pielou's Evenness were calculated using plyr v. 1.8.6 (Wickham, 2020a). The species richness indices Menhinick's, Margalef's were calculated using plyr v. 1.8.6 (Wickham, 2020a). Beta diversity was measured using a Bray–Curtis dissimilarity analysis on the rarefied dataset. The model was tested for statistical significance using a PERMANOVA using the ‘adonis2()’ function in vegan. The relative abundance (%) of each taxa was calculated by dividing the count of each taxa per sample by the total count of all taxa per sample. Significant differences in relative abundance was calculated using an ANOVA (aov(rel_ab~group+Genus)) and p‐values were adjusted using Tukey's HSD. All plots were generated using ggthemes v. 4.2.0 (Arnold, 2019), ggplot 2 v. 3.3.2 (Wickham, 2011), ggpubr v. 0.4.0 (Kassambara, 2020), scales v. 1.1.1 (Wickham & Seidel, 2020), and cowplot v. 1.1.0 (Wilke, 2020). For all microbial data analyses: ad libitum day 14 (*n* = 5), ad libitum day 35 (*n* = 6), ad libitum day 105 (*n* = 6), weight cycling day 14 (*n* = 7), weight cycling day 35 (*n* = 7), and weight cycling day 105 (*n* = 6). RMarkdown code available upon request.

## AUTHOR CONTRIBUTIONS

**Madeline Wight:** Funding acquisition; investigation. **Chequita N. Brooks:** Formal analysis; investigation. **Clare H. Scott Chialvo:** Investigation. **Crystal A. West:** Conceptualization; funding acquisition; project administration; resources. **Aline M. A. de Souza:** Conceptualization; formal analysis; funding acquisition; investigation. **Rachel M. Bleich:** Conceptualization; funding acquisition; project administration; resources.

## CONFLICT OF INTEREST STATEMENT

The authors declare no conflict of interest.

## Supporting information


Data S1.

## Data Availability

The raw data for the 16S rRNA gene sequence has been deposited in the NCBI BioProject database under accession number: PRJNA1274445.
