# Supplementary material for: The impact of weight cycling on gut microbiome richness and diversity in female rats
Source: Physiol Rep. 2026 Mar 24;14(6):e70828. doi: 10.14814/phy2.70828 (PMC13097409; doi:10.14814/phy2.70828)
Supplement: Supplementary file 1 — Data S1. [file PHY2-14-e70828-s001.docx]

**Title:** The Impact of Weight Cycling on Gut Microbiome Richness and Diversity

**Running Title:** Weight Cycling Diet Microbes

**Authors:** ^1^Madeline Wight^†^, ^1,3^Chequita N. Brooks^†^, ^1^ Clare Scott-Chialvo, ^1^Crystal A. West, ^2^ Aline M. A. de Souza^#&^, ^1^ Rachel M. Bleich^#&^

^1^ Department of Biology, Appalachian State University, Boone, NC, USA

^2^ Department of Medicine, Georgetown University, Washington, DC, USA

^3^ Louisiana Universities Marine Consortium, Chauvin, LA, USA

^†^ Co-First Authors

^&^Co-Senior Authors

**^#^Corresponding Authors:**

Rachel M. Bleich

Assistant Professor, Biology

Appalachian State University

Rankin Science West 263

572 Rivers St.

Boone NC 28608

**Email:** [bleichrm@appstate.edu](mailto:bleichrm@appstate.edu)

Aline M.A. de Souza

Assistant Professor, Medicine

Georgetown University

Building D

4000 Reservoir Rd NW

Washington DC 20007

**Email:** [aa1617@georgetown.edu](mailto:aa1617@georgetown.edu)

**ORCID IDs:**

Chequita Brooks - 0000-0002-7684-6470

Rachel Bleich - 0000-0002-8170-483X

Crystal West - 0000-0002-1744-1537

Clare Scott-Chialvo - 0000-0002-9029-3593

Aline de Souza - 0000-0002-7031-2523

**Key Words:** Diet Restriction, Weight Cycling, Gut Microbiome, Microbial Diversity, Food Restriction

**Supplementary Figures.**


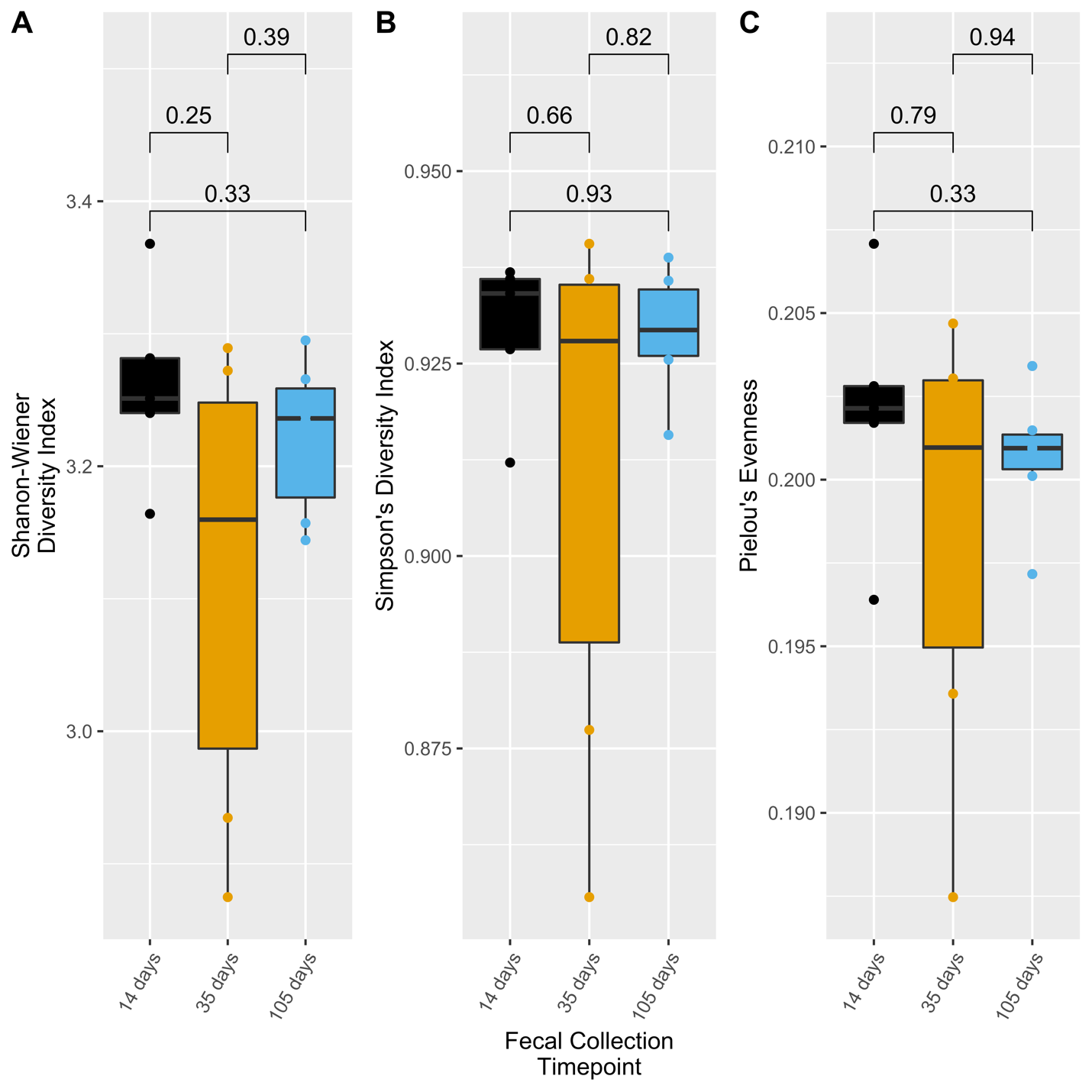


Figure S1. The *ad libitum* treatment group of Fischer F344 rats’ fecal microbiomes were quantified for alpha diversity at three timepoints, days 14, 35, and 105. All rats in this treatment group were fed *ad libitum* for the entire experimental period. Alpha diversity was measured using the Shannon-Wiener diversity index (A) and Simpson’s diversity index (B) and evenness was measured using the Pielou’s evenness index (C). There was no significant difference observed for any index during the experimental period.


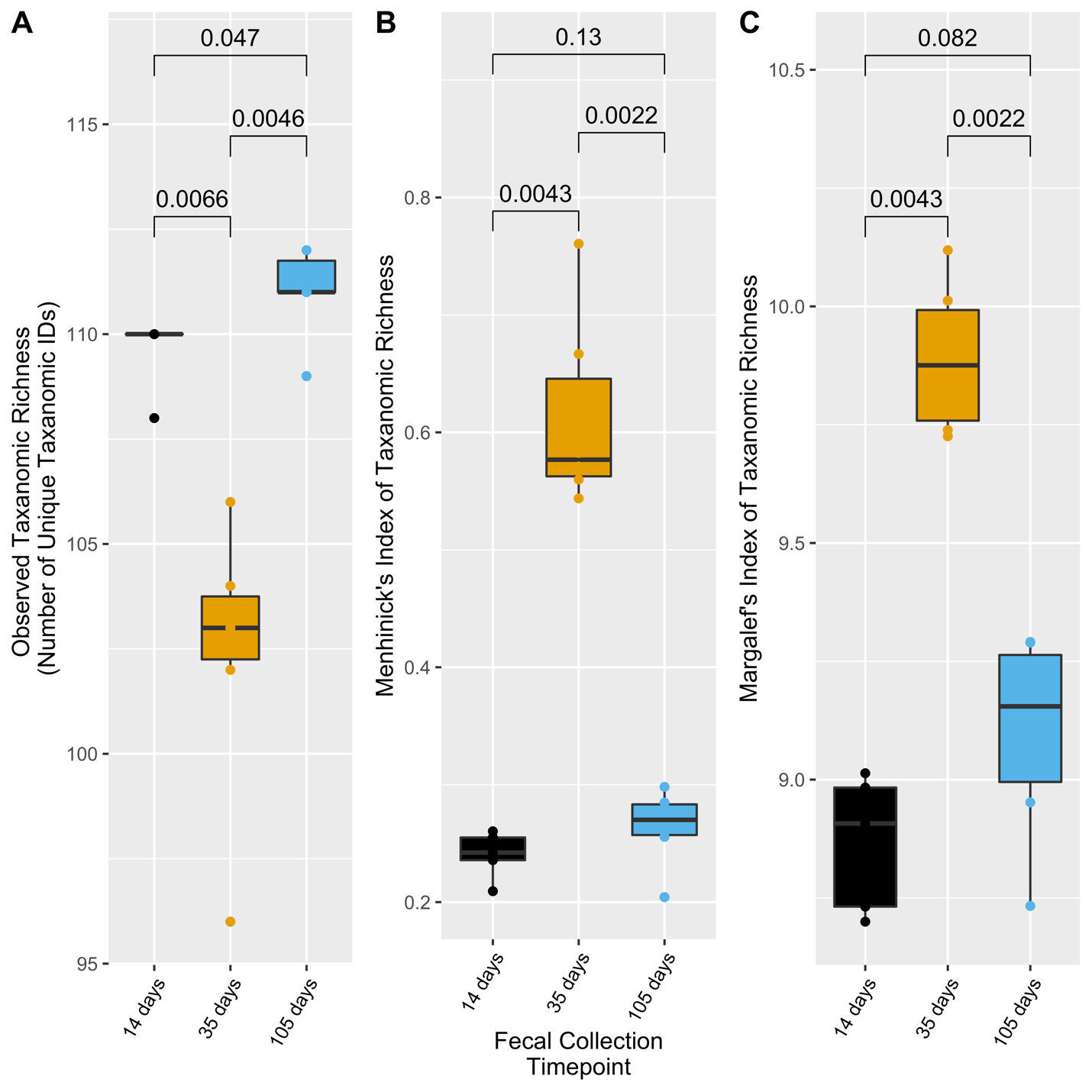


Figure S2. The *ad libitum* treatment group of Fischer F344 rats’ fecal microbiomes were quantified for species richness at three timepoints, days 14, 35, and 105. All rats in this treatment group were fed *ad libitum* for the entire experimental period. Species richness was quantified using observed taxonomic richness (A), Menhinick’s index (B), and Margalef’s index (C). There was no significant difference between days 14 and 105 in the *ad libitum* treatment group microbiomes. There was a significant increase in species richness observed between days 14 and 35 (Menhinick’s; p=0.0043, Margalef’s; p=0.0043) and a significant decrease between days 35 and 105 (Menhinick’s; p=0.0022, Margalef’s; p=0.0022).

**Appendix S1. awk commands to clean up the read-assignment-distributions.tsv file from EMU**

#change tsv to csv

awk 'BEGIN { FS="\t"; OFS="," } {$1=$1; print}' read-assignment-distributions.tsv > read-assignment-distributions.csv

#substitute underscore with separator value between libX_LX_XX and asvX

awk 'NR==1{$1="emu_library,ASV"$1} NR!=1{split($1,a,"_");$1=a[1]"_"a[2]"_"a[3]","a[4];}1' read-assignment-distributions.csv > read-assignment-distributions-asv.csv

#choose the maximum value in each asv assignment row

cat row_max.awk

BEGIN {FS=",";OFS=",";print"emu_library,ASV,tax_id,tax_id2"}

NR == 1 {

for (i=1; i<=NF; i++) headers [i]=$i;

next

}

{

#find maximum value

max=$3

for (i=4;i<=NF;i+=1) if ($i>max) max=$i;

#print row id

printf "%s", $1

sep=OFS

#print asv id

printf "%s%s", sep, $2

#print all column headers of the max value columns

sep=OFS

for (i=3; i<=NF; i+=1) {

if($i==max){

printf "%s%s%s", sep, headers[i], sep;

sep=","

}

}

printf"\n"

}

END {}

awk -f ../row_max.awk read-assignment-distributions-asv.csv > read-assignment-distributions-max.csv

#this output gives the total number of ASVs per lane

awk 'BEGIN{OFS=",";FS=","; print "lane,asv_count"} NR>1{names[$2,$1]=$1} END{for(n in names) lane[names[n]]++; for(l in lane) print l, lane[l]}' read-assignment-distributions-max.csv > 24_03_19_asv_count.csv

#this output gives the total number of assigned taxa per lane

awk 'BEGIN{OFS=",";FS=","; print "lane,number_taxa"} NR>1{names[$3,$1]=$1} END{for(n in names) lane[names[n]]++; for(l in lane) print l, lane[l]}' read-assignment-distributions-max.csv > 24_03_19_taxa_count.csv

#count the number of taxa per lane and record that count in a new .csv w/ column headers lane, tax_id, and count

awk 'BEGIN{OFS=",";FS=","; print "lane,tax_id,count"} NR!=1{a[$1 FS $3]++} END{for(i in a){print i,a[i]}}' read-assignment-distributions-max.csv >24_03_19_taxa_count_indiv.csv

#sanity check, the number of taxa counted sum to the total number of asvs for lane

cat 24_03_19_taxa_count_indiv.csv | grep "lib2_L2_01" | cut -d, -f3 | paste -s -d+ - | bc
